# Postnatal Survival of Mice with Maternal Duplication of Distal Chromosome 7 Induced by a *Igf2*/*H19* Imprinting Control Region Lacking Insulator Function

**DOI:** 10.1371/journal.pgen.1000803

**Published:** 2010-01-08

**Authors:** Li Han, Piroska E. Szabó, Jeffrey R. Mann

**Affiliations:** 1 Division of Biology, Beckman Research Institute, City of Hope National Medical Center, Duarte, California, United States of America; 2 Department of Zoology, The University of Melbourne, Melbourne, Victoria, Australia; 3 Laboratory and Community Genetics Theme, Murdoch Childrens Research Institute, The Royal Children's Hospital, Parkville, Victoria, Australia; University of Cambridge, United Kingdom

## Abstract

The misexpressed imprinted genes causing developmental failure of mouse parthenogenones are poorly defined. To obtain further insight, we investigated misexpressions that could cause the pronounced growth deficiency and death of fetuses with maternal duplication of distal chromosome (Chr) 7 (MatDup.dist7). Their small size could involve inactivity of *Igf2*, encoding a growth factor, with some contribution by over-expression of *Cdkn1c*, encoding a negative growth regulator. Mice lacking *Igf2* expression are usually viable, and MatDup.dist7 death has been attributed to the misexpression of *Cdkn1c* or other imprinted genes. To examine the role of misexpressions determined by two maternal copies of the *Igf2*/*H19* imprinting control region (ICR)—a chromatin insulator, we introduced a mutant ICR (ICR^Δ^) into MatDup.dist7 fetuses. This activated *Igf2*, with correction of *H19* expression and other imprinted transcripts expected. Substantial growth enhancement and full postnatal viability was obtained, demonstrating that the aberrant MatDup.dist7 phenotype is highly dependent on the presence of two unmethylated maternal *Igf2*/*H19* ICRs. Activation of *Igf2* is likely the predominant correction that rescued growth and viability. Further experiments involved the introduction of a null allele of *Cdkn1c* to alleviate its over-expression. Results were not consistent with the possibility that this misexpression alone, or in combination with *Igf2* inactivity, mediates MatDup.dist7 death. Rather, a network of misexpressions derived from dist7 is probably involved. Our results are consistent with the idea that reduced expression of *IGF2* plays a role in the aetiology of the human imprinting-related growth-deficit disorder, Silver-Russell syndrome.

## Author Summary

Parthenogenetic mouse embryos with two maternal genomes die early in development due to the misexpression of imprinted genes. To gain further insight into which misexpressions might be involved, we examined some of the misexpressions that could determine the small size and fetal death of a “partial parthenogenone”—embryos with maternal duplication of distal Chr 7 (MatDup.dist7). We investigated the involvement of two maternal copies of the *Igf2*/*H19* imprinting control region (ICR), which is associated with lack of activity of the *Igf2* gene, encoding a growth factor, and over-activity of *H19*. By introducing a mutant ICR, we activated *Igf2* and expected to correct other misexpressions, such as that of *H19*. The result was substantial increase in growth and full postnatal viability of MatDup.dist7 fetuses, demonstrating the dependency of their abnormal phenotype on two maternal copies of the ICR. Activation of *Igf2* was probably the main effector of this rescue. These results are consistent with the idea that reduced expression of *IGF2* is causal in the human growth deficit disorder, Silver-Russell syndrome.

## Introduction

Parthenogenetic mouse embryos usually die before 6½ days post coitum (dpc). Occasionally they develop to the 25 somite forelimb bud stage or approximately 9½ dpc [1]–[5]. Parthenogenones possess two maternally-derived genomes and would be expected to possess abnormal levels of transcript of all known imprinted genes, that is, lack of expression of paternally expressed genes (two inactive copies), and over-expression of maternally expressed genes (two active copies). Their death is likely a composite effect of at least some of these misexpressions, although those involved are not well defined. Defining the causes is important for improving understanding of the aetiology of genomic imprinting [6]–[9] and the prevalence of sexual reproduction, which ‘has long been an evolutionary enigma’ [10].

Knowledge of the causes of parthenogenetic death has come from two sources. First, the union of unbalanced complementary gametes in intercrosses of mice carrying reciprocal or Robertsonian translocations yield, at low frequency, embryos with maternal duplication and paternal deficiency for particular Chr regions as defined by the translocation breakpoint [11]–[13]. Maternal duplication of twelve Chr regions results in developmental anomalies. Only three of these are associated with peri- or prenatal death, these being maternal duplication of proximal Chr 6 (MatDup.prox6)—prior to 11½ dpc [14], maternal duplication of distal Chr 7 (MatDup.dist7)—late fetal death [15], and maternal disomy of Chr 12—perinatal death, probably attributable to the distal region [16]. Second, knockouts of imprinted genes and imprinting control regions (ICRs) have provided information on the effects of disregulation of imprinted genes, for example, [17]–[21]. To better define the causes of failed parthenogenetic development, and learn more of how imprinted genes at dist7 work together to regulate normal development, we have examined some of the misexpressions of imprinted genes thought to contribute to the abnormal development of MatDup.dist7 conceptuses. These display a pronounced growth deficit of the fetus and placenta and die at the late fetal stage, or possibly at birth. Live MatDup.dist7 young have never been observed [13],[15] (J. Mann, unpublished data).

Dist7 is an important region in terms of genomic imprinting, containing over 20 imprinted genes [13],[22]. At least three of these are regulated by the *Igf2*/*H19* imprinting control region (ICR), these being ‘insulin like growth factor 2’ (*Igf2*)—paternally expressed and encoding a mitogen important for embryonic growth [23],[24], ‘insulin II’ (*Ins2*)—paternally expressed in yolk sac [25], and the non-coding ‘*H19* fetal liver mRNA’ (*H19*) gene—maternally expressed [26]. Other non-coding transcripts have been described, these being *Mir483*, contained within an intron of *Igf2* [27] and for which imprinting status is unknown, *Mir675*, contained with an *H19* exon and therefore likely to follow the imprinting pattern of the host gene [28],[29], and antisense transcripts within *Igf2* [30]. The targets of the *Mir483* and *Mir675* miRNAs are unknown. The maternally-derived *Igf2* allele is inactive due to the hypo-methylated maternal *Igf2*/*H19* ICR functioning as a‘CCCTC-binding factor’ (CTCF)-based chromatin insulator. This lies between the *Igf2* promoter and the shared *Igf2*-*H19* enhancers, preventing their interaction. The maternal *H19* promoter lies on the same side of the insulator as the enhancers, therefore interaction occurs. On the paternal Chr the ICR is hyper-methylated, preventing CTCF binding and insulator formation and allowing for paternal *Igf2* promoter and enhancer interaction. The paternal *H19* promoter, just distal to the methylated ICR, also becomes methylated, and is inactive. The *Ins2* gene is located just distal to *Igf2*. The *Ins2* parental alleles are affected in the same way as their *Igf2* counterparts, but only in yolk sac. *Ins2* is expressed biallelically in pancreas [25], [31]–[33].

Telomeric or distal to the *Igf2*/*H19* ICR domain is a large cluster of imprinted genes under regulatory control of the Kv differentially methylated region (DMR)-1 (KvDMR1) ICR. The active state of maternally-derived genes within this cluster is coincident with maternal-specific ICR methylation and the inactive state of the promoter of the ‘KCNQ1 overlapping transcript 1’ (*Kcnq1ot1*) gene contained within the ICR. The paternal ICR is hypo-methylated, and paternal-specific elongation of the *Kcnq1ot1* transcript is coincident with silencing in cis of genes within the cluster [17],[34],[35]. One of the genes regulated by this ICR is the ‘cyclin-dependent kinase inhibitor 1C (P57)’ (*Cdkn1c*) gene encoding a protein facilitating reduced cell proliferation, increased apoptosis and delayed cell differentiation [36],[37].

MatDup.dist7 fetuses are maternally duplicated for the hypo-methylated *Igf2*/*H19* ICR and hyper-methylated KvDMR1 ICR regions, as well as for other imprinted transcripts at dist7. This epigenetic configuration is highly similar to that associated with the human imprinting-related growth deficit disorder, Silver-Russell syndrome (SRS) (OMIM 180860). More than half of cases are associated with hypo-methylation of the *IGF2*/*H19* ICR, also known as ‘ICR1’. The disease is also associated with maternal duplication of the KvDMR1 ICR region, also known as ‘ICR2’, and maternal duplication of the 11p15.5 Chr region encompassing both ICRs. It is strongly suspected that SRS is caused by downregulation of *IGF2*, and, in a minority of cases, excess *CDKN1C* or other imprinted genes regulated by ICR2. However, empirical evidence is lacking [38]–[40].

The death of MatDup.dist7 fetuses has been difficult to decipher. Available evidence suggests that maternal duplication of the *Igf2*/*H19* ICR regulatory domain alone is insufficient to explain the total phenotype observed. Mice with paternal inheritance of a tandem duplication of a chicken β-globin CTCF-based chromatin insulator, substituted for the endogenous *Igf2*/*H19* ICR, are similar to MatDup.dist7 mice in having a fully functional hypo-methylated insulator on both parental Chrs. They lack *Igf2* activity, have at least twofold over-expression of *H19*, with both parental alleles probably active, and would be expected to lack *Ins2* activity in yolk sac. Nevertheless, their phenotype—dwarfism combined with postnatal viability—is essentially identical to *Igf2* mutants [41]. Mice homozygous for this genetic modification, in a mix of strains 129S1/SvImJ and outbred Swiss CF-1, showed normal fecundity and were maintained as a random-bred line for several years (J. Mann, unpublished data). Further, lack of *Igf2* activity is unlikely to be the sole cause of reduced growth in MatDup.dist7 fetuses. At 17½ dpc, their weight is approximately 40% of wild-type [42] (J. Mann and Walter Tsark, unpublished observations) compared to 50–60% of wild-type for *Igf2* mutants and mice maternally inheriting the chicken insulator [41]. Overall, these observations indicate that the MatDup.dist7 phenotype of fetal growth deficit and death involves the misexpression of imprinted genes outside the influence of the *Igf2*/*H19* ICR, and this has previously been suggested [42].

Available evidence also indicates that maternal duplication of the KvDMR1 ICR regulatory domain alone is insufficient to explain the total phenotype observed. Mice with paternal inheritance of a deletion of this element exhibit biallelic expression of adjacent imprinted genes. These mice, in a mix of mouse strains 129S4/SvJae and C57BL/6J, are postnatally viable. They show some reduction in size, and it has been indicated that this is caused by over-expression of *Cdkn1c* [35]. Reduced growth has also been observed in *Cdkn1c*-BAC transgenic mice. While these displayed high frequency perinatal mortality in strain 129/Sv, high postnatal viability was obtained in a mix of strains 129/Sv and outbred Swiss MF1 [43]. These observations indicate that MatDup.dist7 late fetal death, occurring in the context of mixed strains including outbred Swiss, involves the misexpression of imprinted genes outside the influence of the KvDMR1 ICR. Overall, these observations have led to suggestions that MatDup.dist7 death could be a composite effect of misexpressions derived from both imprinted domains, for example, *Igf2* inactivity combined with *Cdkn1c* over-expression [43].

To define the role of imprinted genes regulated by the *Igf2*/*H19* ICR in the MatDup.dist7 phenotype, we evaluated the effects of introducing a mutated *Igf2*/*H19* ICR (ICR^Δ^) which cannot bind CTCF and form an insulator [44]. MatDup.dist7 fetuses carrying ICR^Δ^ would be expected to be corrected in terms of the number of active alleles of *Igf2*—activation of one of two inactive alleles, *H19*—repression of one of two active alleles, and *Ins2*—activation of one of two inactive alleles in yolk sac. MatDup.dist7 fetuses carrying ICR^Δ^ were significantly rescued in terms of growth and were able to survive to adulthood. These results demonstrate that the aberrant phenotype of MatDup.dist7 fetuses is highly dependent on the presence of two maternally-derived *Igf2*/*H19* ICR chromatin insulators.

## Results

### Maternal Inheritance of ICR^Δ^ Rescues Growth in *Igf2* Null Mutants

Maternal inheritance of ICR^Δ^ results in activation of *Igf2* in cis such that total *Igf2* RNA is 1.7 and 2.1 times the normal level in the liver and kidney of 17½ dpc fetuses, respectively, and also repression of *H19* in cis, such that total *H19* RNA is 0.2 and 0 times the normal level in these same tissues, respectively [44]. This configuration of expression—two active *Igf2* and two inactive *H19* alleles—is coincident with increased growth, an effect thought to be due to the former misexpression [18],[45],[46]. Lack of *H19* RNA alone has no effect on *Igf2* expression or imprinting and results in no discernible phenotype [47]. Maternal inheritance of ICR^Δ^ would also be expected to result in activation of *Ins2* in yolk sac.

**Figure 1:**
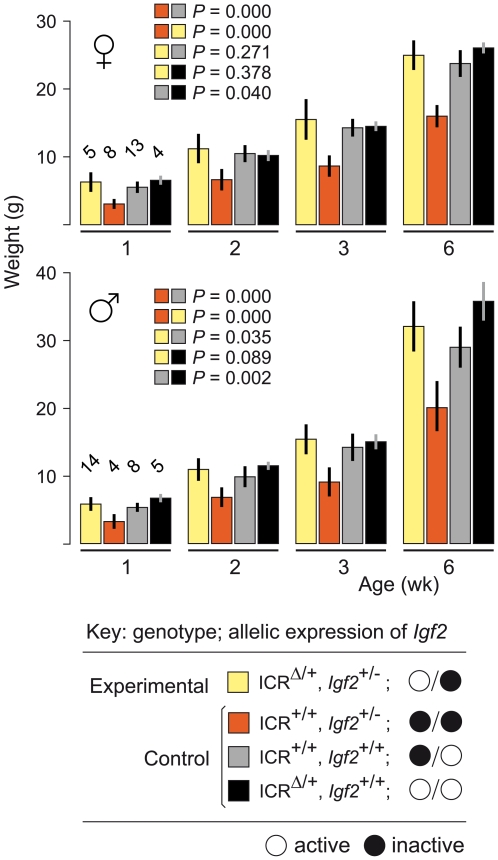
Weight gain in *Igf2* mutants carrying ICR^Δ^. Young were obtained from (ICR^Δ/+^, *Igf2* ^+/+^ ♀×ICR^+/+^, *Igf2* ^+/−^ ♂) matings. Bars are mean±s.d., with (n) given above the 1 wk bars. Key: alleles are maternal/paternal in derivation. The two-sample *t*-test was used to determine the probability that two 6 week samples (identified as paired squares) were equal.

To confirm that maternal inheritance of ICR^Δ^ can mediate normal growth, we tested its function in mice paternally inheriting a null mutation of *Igf2* (*Igf2* ^−^). Mice of genotype (ICR^+/+^, *Igf2* ^+/−^) are small due to lack of *Igf2* activity, with the maternal allele inactive, and the paternal allele null [24]. Results are shown in Figure 1. Experimental young of genotype (ICR^Δ/+^, *Igf2* ^+/−^), in which the maternally-derived *Igf2* allele is activated in cis by ICR^Δ^, were not significantly different in weight to control (ICR^+/+^, *Igf2* ^+/+^) mice at 6 weeks of age (females, *P* = 0.271; males, *P* = 0.035). Thus, a single maternal copy of ICR^Δ^ induces sufficient *Igf2* activity for achieving normal postnatal growth. We note that, in respect to growth with one versus two active *Igf2* alleles, experimental (ICR^Δ/+^, *Igf2* ^+/−^) animals with one active allele (maternal), were not significantly different in weight to (ICR^Δ/+^, *Igf2* ^+/+^) animals with two active alleles (females, *P* = 0.378; males, *P* = 0.089). Further, (ICR^+/+^, *Igf2* ^+/+^) females with one active allele (paternal), were not significantly different in weight to (ICR^Δ/+^, *Igf2* ^+/+^) females with two active alleles (*P* = 0.04). However, in males, mice with one active allele (paternal) were lighter than mice with two active alleles, as expected (*P* = 0.002). Given the borderline probability values obtained, greater numbers of animals need to be analysed to accurately determine the relative growth rates of mice of the various genotypes.

### ICR^Δ^ Rescues Growth and Viability in MatDup.dist7 Fetuses

MatDup.dist7 zygotes were produced in intercrosses of mice carrying the reciprocal translocation T(7;15)9H (T9H). Such intercrosses give rise to a high proportion of unbalanced zygotes, and litter size is small. Of balanced zygotes, only one in seven are expected to be MatDup.dist7, these identified by the dist7 marker, albino (*c*), a mutation of the ‘tyrosinase’ (*Tyr*) gene [15].

**Figure 2:**
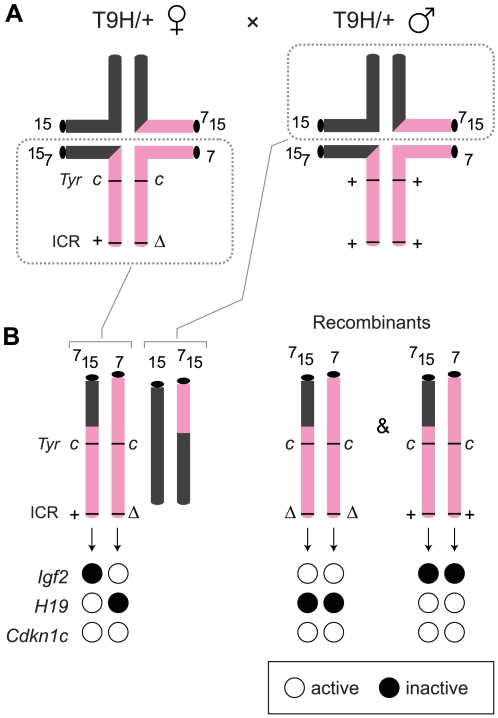
Production of MatDup.dist7 fetuses. (A) Quadrivalents at meiosis I occurring in (T9H/+, *Tyr^c^* ^/*c*^, ICR^Δ/+^ ♀×T9H/+, *Tyr* ^+/+^, ICR^+/+^ ♂) matings. Female reciprocal translocation heterozygote (T9H/+) parent is homozygous for the dist7 marker albino (*Tyr^c^* ^/*c*^) and carries ICR^Δ^ (ICR^Δ/+^), while the male T9H/+ parent is wild-type at both of these loci (*Tyr* ^+/+^, ICR^+/+^). (B) Genotypes of MatDup.dist7 fetuses obtained from the union of unbalanced complementary gametes. MatDup.dist7 individuals are readily identified as albinos from 12½ dpc by lack of eye or fur pigmentation. Many of these are ICR^Δ.+^ although a high frequency of homozygous recombinants, ICR^Δ.Δ^ and ICR^+.+^, are also obtained. Chrs depicted are actually paired chromatids. Underneath is depicted allele-specific expression—MatDup.dist7 ICR^Δ.+^ and ICR^Δ.Δ^ fetuses have one and probably two activated *Igf2* alleles, and should have one and two repressed *H19* alleles, respectively.

**Figure 3:**
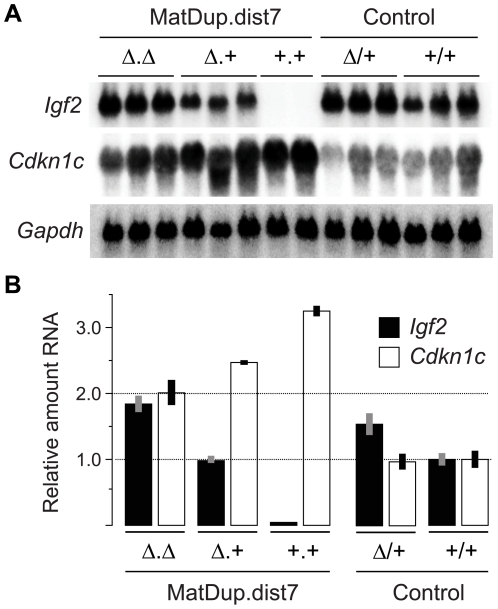
Expression of imprinted genes in 13½ dpc MatDup.dist7 fetuses. (A) Northern blots for the imprinted genes *Igf2* and *Cdkn1c*, and for the housekeeping *Gapdh* gene for normalization. Each lane is an individual fetus. ICR genotype is given immediately above the lanes. (B) Northern blots in (A) were quantitated to show relative RNA levels. Values for *Igf2* and *Cdkn1c* were normalized to *Gapdh* RNA, calibrated to control ICR^+/+^ values, and adjusted to a mean of 1.0. Values are mean±s.d. with (n) as shown in (A). Mating scheme to breed these animals was as described in the legend to Figure 2.

**Figure 4:**
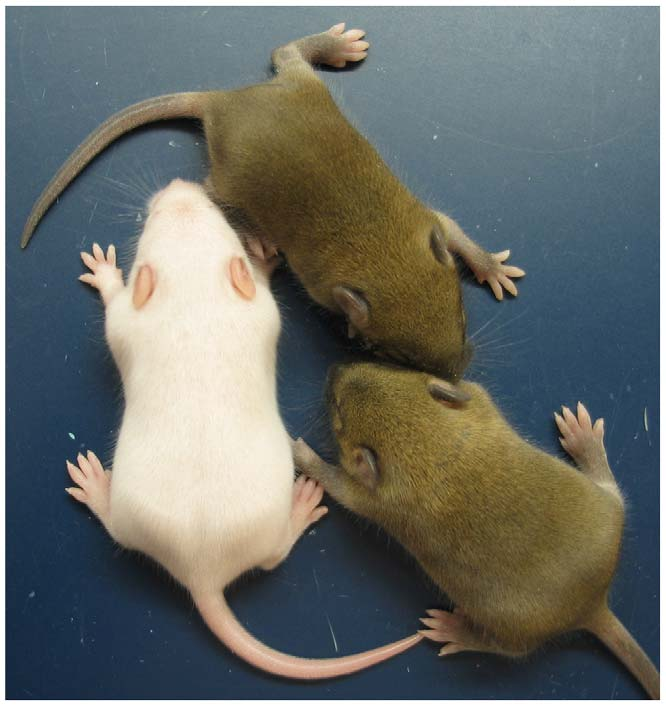
Neonatal MatDup.dist7 mouse rescued by ICR^Δ^. MatDup.dist7 (ICR^Δ.+^, *Cdkn1c* ^+.+^) albino neonate with two agouti littermates at 10 days post-partum. Mating scheme to breed these animals was as described in the legend to Figure 2.

**Figure 5:**
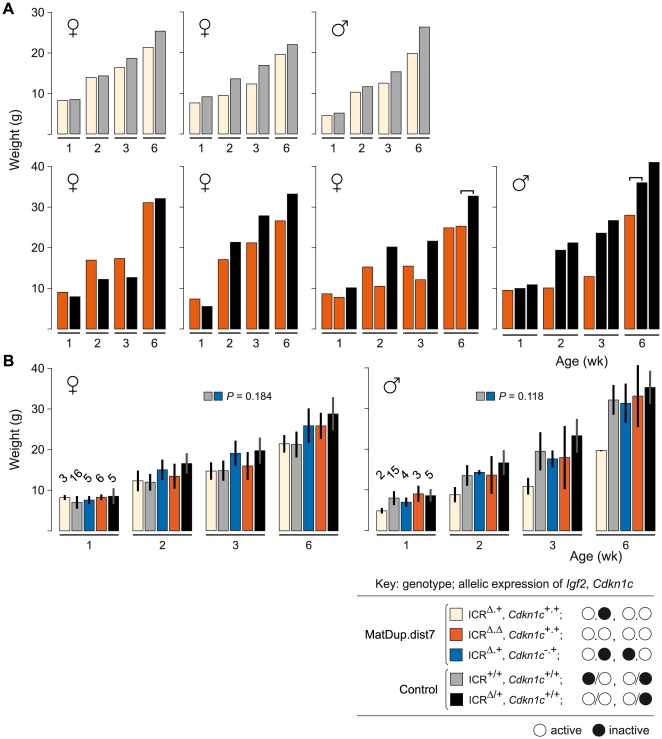
Weight gain in post-partum MatDup.dist7 mice rescued by ICR^Δ^. (A) Weight gain in litter matched MatDup.dist7 (ICR^Δ.+^, *Cdkn1c* ^+.+^) or (ICR^Δ.Δ^, *Cdkn1c* ^+.+^) young obtained from matings of (T9H/+, *Tyr^c^* ^/*c*^, ICR^Δ/+^ ♀×T9H/+, Tyr^+/+^, ICR^+/+^ ♂) and (T9H/+, *Tyr^c^* ^/*c*^, ICR^Δ/+^, *Cdkn1c* ^+/−^ ♀×T9H/+, *Tyr* ^+/+^, ICR^+/+^, *Cdkn1c* ^+/+^ ♂) mice. Each graph represents a single litter where at least one control with the same number of active *Igf2* alleles was obtained. Each bar represents a single animal. The paired-sample *t*-test was used to determine the probability that the weight of MatDup.dist7 and control mice was equal. For three-animal litters, the pairs used in the statistical test are indicated by the bracket above the bars. The *P* value was <0.01 for 6 week old mice, indicating a significant difference, while there was no significant difference in weight at 1, 2, and 3 weeks of age. (B) All weight gain data collected from the two sets of matings as described in (A) regardless of littermate matching. Bars are mean±s.d., with (n) given above the 1 wk bars. The two-sample *t*-test was used to determine the probability that two 6 week samples (identified as paired squares) were equal. Key: alleles are maternal.maternal in derivation for MatDup.dist7 and maternal/paternal in derivation for controls.

The ICR^Δ^ mutation was introduced into female T9H/+ parents and was inherited by MatDup.dist7 zygotes (Figure 2). Expected allelic activity of *Igf2* and *Cdkn1c* in the three possible MatDup.dist7 genotypes is shown (Figure 2B). ICR^Δ^-induced activation of *Igf2* was confirmed in 13½ dpc MatDup.dist7 fetuses obtained in (T9H/+, *Tyr^c^* ^/*c*^, ICR^Δ/+^ ♀×T9H/+, *Tyr* ^+/+^, ICR^+/+^ ♂) intercrosses. The level of *Igf2* transcript in MatDup.dist7 ICR^Δ.+^ fetuses was the same as in control ICR^+/+^ fetuses with one active allele, while it was almost double the normal amount in MatDup.dist7 ICR^Δ.Δ^ fetuses with probably two active alleles (Figure 3A and 3B). Increased total *Igf2* RNA was also seen in mice which maternally inherit ICR^Δ^ and have an active maternal and paternal allele of *Igf2* (Figure 3A and 3B). Also, MatDup.dist7 fetuses of all genotypes contained at least double the amount of *Cdkn1c* RNA relative to controls, probably because of two active alleles (Figure 3A and 3B). These intercross matings were allowed to proceed to term and we immediately began to observe viable albino or MatDup.dist7 young which were of overtly similar size to agouti littermates. A MatDup.dist7 animal and its two littermates at 10 days post-partum is shown (Figure 4). All MatDup.dist7 young obtained were of genotype ICR^Δ.+^ or recombinant ICR^Δ.Δ^. Seven of 52 mice born were MatDup.dist7 which is similar to the expected frequency, indicating that ICR^Δ^ was always able to increase growth and rescue viability. In age- and litter-matched animals, a significant weight deficit of approximately 17% in MatDup.dist7 animals became apparent at 6 weeks of age when compared with controls carrying an equivalent number of active *Igf2* alleles, that is, MatDup.dist7 ICR^Δ.+^ with control ICR^+/+^ (one active allele each) and MatDup.dist7 ICR^Δ.Δ^ recombinant with control ICR^Δ/+^ (probably two active alleles each) (Figure 5A).

### Involvement of *Cdkn1c* Expression in MatDup.dist7 Death

CDKN1C may antagonize the growth promoting effects of IGF2 [17],[48], and it has been suggested that excess CDKN1C may combine with lack of IGF2 to cause MatDup.dist7 death [43]. To test this possibility, we introduced a null allele of *Cdkn1c* (*Cdkn1c* ^−^) into MatDup.dist7 fetuses to enforce its monoallelic expression. In (T9H/+, *Tyr^c^* ^/*c*^, ICR^+/+^ *Cdkn1c* ^+/−^ ♀×T9H/+, *Tyr* ^+/+^, ICR^+/+^ *Cdkn1c* ^+/+^ ♂) matings, all of 55 young obtained were agouti controls, that is, at least six albino MatDup.dist7 (ICR^+.+^, *Cdkn1c* ^+.−^) pups were expected, but none were observed. This result is not consistent with the idea that MatDup.dist7 death results only from the combined action of the *Cdkn1c* and *Igf2* misexpressions.

### Involvement of *Cdkn1c* Expression in MatDup.dist7 ICR^Δ.+^ Postnatal Growth Deficit

To test for a role of *Cdkn1c* over-expresssion in the growth deficit at 6 weeks of age of rescued postnatal MatDup.dist7 ICR^Δ.+^ animals, we introduced *Cdkn1c* ^−^ into MatDup.dist7 fetuses such that they were of genotype (ICR^Δ.+^, *Cdkn1c* ^+.−^). This genotype should be normalized for the number of active alleles of imprinted genes regulated by the *Igf2*/*H19* ICR, and also be normalized for *Cdkn1c* expression, that is, all of these imprinted genes should be monoallelically expressed. In (T9H/+, *Tyr^c^* ^/*c*^, ICR^Δ/+^, *Cdkn1c* ^+/−^ ♀×T9H/+, *Tyr* ^+/+^, ICR^+/+^, *Cdkn1c* ^+/+^ ♂) matings, viable MatDup.dist7 ICR^Δ.+^, *Cdkn1c* ^+.−^ young were obtained and these did not display a significant weight deficit at 6 weeks of age—with the caveat that the weight measurements are relative to control young obtained in the previous matings (Figure 5B). Their weights could not be compared to littermates as, given the mating scheme, agouti littermates were always positive for ICR^Δ^—inheritance of *Cdkn1c* ^−^ being lethal—and therefore possessed two active copies of *Igf2*. In any event, these results are consistent with the possibility that biallelic expression of *Cdkn1c* does contribute to a reduction in postnatal growth in MatDup.dist7 ICR^Δ.+^ or ICR^Δ.Δ^, *Cdkn1c* ^+.+^ animals.

## Discussion

We have shown that maternal introduction of a mutant *Igf2*/*H19* ICR, which lacks chromatin insulator activity, into MatDup.dist7 fetuses substantially alters their abnormal phenotype—small size and death at the late fetal stage—to one of near normal growth rate and survival to adulthood. This result clearly demonstrates the dependence of this phenotype on a misexpression of imprinted genes caused by the presence of two active maternally-derived *Igf2*/*H19* ICR chromatin insulators. As this ICR is known to regulate the expression of at least three dist7 imprinted genes—*H19*, *Ins2*, *Igf2*, and a number of non-coding transcripts—correction in the misexpression of one or more of these was probably responsible for the result obtained. Activation of *Igf2* was likely an important correction, this being the only alteration in expression induced by ICR^Δ^ expected to affect growth.

The survival of MatDup.dist7 mice with ICR^Δ^ is more difficult to decipher. As discussed in the Introduction section, it is unlikely that the *Igf2*/*H19* ICR-derived misexpressions are solely responsible for their death, as mice with two functional chromatin insulators—a maternally-derived *Igf2*/*H19* ICR, and a paternally-derived chicken insulator substituted for the *Igf2*/*H19* ICR, possess the same combination of misexpressions as MatDup.dist7 mice in respect to this region, yet these animals have normal postnatal viability [41]. Further evidence is provided by observations of the effects of misexpression of each imprinted gene alone. First, for *H19*, no overt effect on phenotype is observed in transgenic mice with ectopic over-expression [49]–[52]. Biallelic or over-expression of *H19* has been suggested to cause perinatal death of mice produced by combining a non-growing oocyte genome (ng), carrying a deletion of the distal Chr 12 IG-DMR ICR (Δ12), with a fully grown oocyte genome (fg)—ngΔ12/fg mice [53]. However, these mice would be predicted to have the equivalent expression profile of imprinted genes as mice with maternal inheritance of the chicken insulator substitution. The latter mice are viable, despite twofold over-expression of *H19* [41]. Therefore, the perinatal death of ngΔ12/fg mice may result from the combined action of *H19* RNA excess—or possibly *Igf2* RNA absence—and small imperfections in expression derived from the non-growing oocyte genome, for example, as related to the IG-DMR ICR deletion. Second, for *Ins2*, mice lacking in expression of this gene are viable [54]. Third, for *Igf2*, mice lacking expression are dwarfed and have impaired lung development [55], but are usually viable. High postnatal survival frequency of *Igf2* mutants is seen in inbred strain 129/SvEv [23],[24] although in this strain we have observed a low level of perinatal death (J. Mann, unpublished observations). In the present study, in a mix of strains 129/SvEv and outbred Swiss CF-1, we observed high frequency survival. Also, in this same strain mix, we maintained a *Igf2* ^−/−^ random-bred line for a number of years which had normal fecundity (J. Mann, unpublished data). On the other hand, use of a second *Igf2* null mutation [56] revealed that lack of IGF2 in strain C57BL/6J results in death at birth. This effect was not peculiar to this second knockout allele as homozygous mutants can be obtained in strain 129 (M. Constancia, personal communication). In the present study, MatDup.dist7 young were a mix of strains 129S1/SvImJ, CF-1, C57BL/6J and CBA/Ca. In this mix, lack of *Igf2* activity is highly likely to be compatible with survival. Given these various lines of evidence, the present experiments strongly suggest that misexpressed imprinted genes, as regulated by the *Igf2*/*H19* ICR, work in combination with misexpressions derived outside of this region of influence in causing the total MatDup.dist7 phenotype.

The significant rescue in growth probably mediated by *Igf2* activation may also be directly related to MatDup.dist7 survival in that it could compensate for negative effects derived from outside the *Igf2/H19* ICR region. Nevertheless, we cannot rule out the possibility that *Ins2* inactivity in yolk sac, excess *H19* RNA, or the misexpression of non-coding RNAs regulated by the *Igf2*/*H19* ICR make a contribution to the lethal effect. These possibilities could be investigated through correction of their misexpression in MatDup.dist7 fetuses, then determining growth and survival. For example, correction of *H19* over-expression could be achieved by introducing a deletion of the transcript region only.

The imprinted genes operating outside the influence of the *Igf2*/*H19* ICR that contribute to MatDup.dist7 death would be expected to require maternal-, rather than paternal-specific imprinting or methylation for attaining differential expression in the normal context. This is because for full-term development, there is apparently no other requirement, aside from *Igf2*/*H19* ICR methylation, for paternal imprinting at dist7 [57]. The cluster of genes requiring maternal-specific methylation of the KvDMR1 ICR for activity fulfills this criterion. While the introduction of a null mutation of *Cdkn1c*, and hence enforced monoallelic expression of this gene, did not rescue MatDup.dist7 fetuses, this does not rule out the possibility that CDKN1C excess has a role in causing MatDup.dist7 death. In MatDup.dist7 (ICR^+.+^, *Cdkn1c* ^+.+^) fetuses, *Cdkn1c* RNA levels were found to be more than three times that of controls, suggesting that each maternally-derived *Cdkn1c* allele was upregulated 1.5-fold. Therefore, CDKN1C could still be in excess in MatDup.dist7 (ICR^+.+^, *Cdkn1c* ^+.−^) animals. Also, there remains the possibility that excess *Cdkn1c* RNA may contribute as part of a network of misexpressions derived from the cluster regulated by the KvDMR1 ICR. For example, biallelic expression of the ‘pleckstrin homology-like domain, familiy A, member 2’ (*Phlda2*) gene results in placental growth retardation and marginal fetal growth restriction [58], and upregulation of *PHLDA2* is correlated with growth retardation in humans [59],[60]. Also, it has been suggested that excess expression of the ‘achaete-scute complex homolog 2 (*Drosophila*) (*Ascl2*) gene could cause the MatDup.dist7 lethal effect [42]. The phenotype of MatDup.dist7 fetuses could also involve misexpressions of dist7 imprinted genes lying outside of the influence of the two known ICRs. For example, ‘adenosine monophosphate deaminase 3’ (*Ampd3*)—maternally expressed in placenta, and identified in a transcriptome analysis of MatDup.dist7 conceptuses [22], ‘inositol polyphosphate-5-phosphatase F’ (*Innp5f*)—an isoform paternally expressed in brain [61], and ‘cathepsin D’ (*Ctsd*)—possible paternal-specific expression [62].

The postnatal weight deficit of approximately 17% in MatDup.dist7 young at 6 weeks of age was similar to that in mice paternally inheriting a deleted KvDMR1 ICR. This deletion results in biallelic expression of imprinted genes regulated by this ICR, including *Cdkn1c* [17],[34]. Indeed, excess CDKN1C has been indicated as the cause of the weight deficit [35]. Consistent with this possibility is that the weight of MatDup.dist7 ICR^Δ.+^, *Cdkn1c* ^+.−^ young was normal at 6 weeks of age. However, we note that MatDup.dist7 neonates displayed no significant weight deficit until reaching adulthood, while in mice paternally inheriting the deleted KvDMR1 ICR, the weight deficit is present in fetuses and persists throughout postnatal development [17]. More data regarding weight gain in relation to the inheritance of ICR^Δ^, in MatDup.dist7 young and otherwise, is required to confirm these observations.

In terms of MatDup.dist7 death, additional experiments are required to determine exactly which combination of misexpressions are involved. The total MatDup.dist7 phenotype has been ascribed to the very distal portion of Chr 7 as defined by the reciprocal translocation T(7;11)65H (T65H) [42]. This translocation has a breakpoint far more distal on Chr 7 relative to the T9H translocation used in this study, although is still proximal to the two clusters of imprinted genes regulated by the *Igf2*/*H19* and KvDMR1 ICRs. However, some caution should be exercised in ascribing the total effect to this region. While it was shown that T65H- and T9H-MatDup.dist7 fetuses are of similar morphology [42], the postnatal viability of the former was not investigated. If T65H-MatDup.dist7 fetuses are also inviable, then the composite lethal effect is likely to be contained within the two aforementioned clusters of imprinted genes. Evidence that the KvDMR1 cluster contributes to the effect could be obtained by determining the viability of MatDup.dist7 fetuses carrying a deletion of this whole cluster. This would result in enforced monoallelic expression of all genes under regulation of the KvDMR1 ICR, including *Cdkn1c*, and these mice and would be expected to be postnatally viable, although small because of *Igf2* inactivity. Such a deletion, made through truncation of Chr 7 at a point distal to the *Ins2* gene, has been described [63]. A complication with this possible experiment is the existence of imprinted genes at dist7 which are not regulated by either ICR. Another experiment could be to breed mice with paternal inheritance of the chicken β-globin insulator substituted for the *Igf2*/*H19* ICR [41] combined with paternal inheritance of the KvDMR1 ICR deletion [17]. These would misexpress all imprinted genes under regulatory control of both ICRs. If these were the only misexpressions involved in the MatDup.dist7 phenotype, then the phenotype should be reproduced.

MatDup.dist7 fetuses provide an epigenetic model of a subtype of human Silver-Russell syndrome (SRS) involving maternal duplication of the orthologous Chr region, 11p15.5, which encompasses ICR1 and ICR2. In these fetuses, we have shown that abrogation of ICR1 insulator function was able to restore *Igf2* expression, concomitant with restoration of growth and survival. The most common subtype of SRS, that involving hypo-methylation of ICR1, is perhaps better modelled in mice maternally inheriting the chicken insulator in place of ICR1. These animals provide information on the effects of the presence of two functional insulators at the *Igf2*/*H19* region as the only epigenetic lesion. In these fetuses, we previously showed that DNA methylation was abrogated while insulator function remained intact. This resulted in reduced *Igf2* activity and growth retardation [41]. Both of these findings support the idea that reduced expression of *IGF2* during fetal development is causal in the development of SRS. They also support the suggestion that the failure to detect low concentrations of serum IGF2 in SRS patients is related to downregulation of *IGF2* by this stage [38]. Further genetic manipulation in these mouse models should provide additional implications for the human disease.

Our experiments suggest that misexpression of imprinted genes caused by two maternal copies of the *Igf2*/*H19* ICR constitute one component of a composite barrier to parthenogenetic development that was not previously predicted. The lethal effect in MatDup.dist7 fetuses may be specific to later stages of development, and may not normally occur in parthenogenones given their peri-implantation death. Nevertheless, high-level paternal- and maternal-specific expression of *Igf2* and *H19*, respectively, is present shortly after implantation, at least by 6½ dpc [64]. Therefore, it cannot be ruled out that these misexpressions, and others regulated by the *Igf2*/*H19* ICR, play a role in what probably is a complex composite lethal effect involving a network of misexpressed imprinted genes. Indeed, the fact that parthenogenones fail earlier in development than embryos with maternal duplication of any single Chr region, indicates that misexpressions of imprinted genes from different regions are cumulative or synergistic in their deleterious effects. Further, at the molecular level, it has been shown that disregulation of the imprinted genes ‘pleiomorphic adenoma gene-like 1’ (*Plagl1*) and *H19* can affect the expression of other imprinted genes in an imprinted gene expression network [65],[66].

Previous observations have shown that the normal activity of imprinted genes regulated by the *Igf2*/*H19* ICR are one of a small number of developmentally critical expression profiles provided exclusively by imprinting through the male germ line, provided that most if not all other imprinted genes are not misexpressed [57]. The present results raise the possibility that full-term parthenogenetic development could be achieved by correcting the misexpressions of only a few imprinted genes in order to repair the total expression network. One necessary correction would be to activate the ‘paternally expressed 10’ (*Peg10*) gene. Lack of expression of this gene results in death by 10½ dpc, and this misexpression alone would be expected to present a barrier to parthenogenesis. It would be expected to contribute to, or could be solely responsible for, the embryonic death of MatDup.prox6 mice, which occurs prior to 11½ dpc [20].

## Materials and Methods

### Mouse Lines

Line no.; genotype; strain; source, how produced, or reference: Line-1; 129S1/SvImJ (129S1); *Tyr* ^+/+^; The Jackson Laboratory, stock no. 002448. Line-2; outbred Swiss CF-1; *Tyr^c^* ^/*c*^; Charles River Laboratories. Line-3; T9H/T9H, *Tyr* ^+/+^; mix of C57BL/6J (B6) and CBA/Ca (CB); The Jackson Laboratory, stock no. 001752. Line-4; T9H/T9H, *Tyr^c^* ^/*c*^; mix of B6, CB and CF-1; made by mating line-2 with -3, then intercrossing. Line-5; *Tyr^c^* ^/*c*^, ICR^Δ/Δ^; mix of CF-1 and 129S1; made by mating ICR^Δ/+^ mice [44] with line-2, then intercrossing. Line-6; *Tyr^c^* ^/*c*^, *Cdkn1c* ^+/−^; mix of 129S7/SvEvBrd (129S7), B6 and CF-1; made by mating *Cdkn1c* ^+/−^ mice [37] with line-2, then intercrossing. Line-7; T9H/T9H, *Tyr^c^* ^/*c*^, *Cdkn1c* ^+/−^; mix of strains B6, CB, CF-1, and 129S7; made by mating line-4 with -6, then intercrossing. Line-8; *Igf2* ^+/−^; 129/SvEv [23].

### Matings

Production of experimental (ICR^Δ/+^, *Igf2* ^+/−^) mice (Figure 1): Female parents (ICR^Δ/+^, *Igf2* ^+/+^) were bred in (line-5 ♀×line-1 ♂) matings. Male parents (ICR^+/+^, *Igf2* ^+/−^) were of line-8. Young were a mix of strains 129 and CF-1. Production of MatDup.dist7 ICR^Δ.+^ and ICR^Δ.Δ^ mice (Figure 2): Female parents (T9H/+, *Tyr^c^* ^/*c*^, ICR^Δ/+^) were bred in (line-5 ♀×line-4 ♂) matings. Male parents (T9H/+, *Tyr* ^+/+^, ICR^+/+^) were bred in (line-3 ♀×line-1 ♂) matings. Young were a mix of strains 129S1, CF-1, B6, and CB. Production of MatDup.dist7 *Cdkn1c* ^−.+^ young, attempted: Female parents (T9H/+, *Tyr^c^* ^/*c*^, *Cdkn1c* ^+/−^) were bred in (line-4 ♀×line-6 ♂) matings. Male parents (T9H/+, *Tyr* ^+/+^, ICR^+/+^) were bred in (line-3 ♀×line-1 ♂) matings. Young were a mix of strains 129, B6, CB, and CF-1. Production of MatDup.dist7 (ICR^Δ.+^, *Cdkn1c* ^+.−^) young (Figure 5B): Female parents (T9H/+, *Tyr^c^* ^/*c*^, ICR^Δ/+^, *Cdkn1c* ^+/−^) were bred in (line-5 ♀×line-7 ♂) matings. Male parents (T9H/+, *Tyr* ^+/+^, ICR^+/+^) were bred in (line-3 ♀×line-1 ♂) matings. Young were a mix of strains 129S1, 129S7, B6, CB, and CF-1.

### Genotyping

For the ICR, two pairs of primers were used. The first pair was specific for the mutant ICR, identical to a pair previously described [41]: 5′- GCCC ACCA GCTG CTAG CCATC -3′ and 5′- CCTA GAGA ATTC GAGG GACC TAAT AAC -3′, 240 bp amplicon identified ICR^Δ.+^ and ICR^Δ.Δ^ animals. The second pair was specific for ICR^+^, with primers binding to sequence positions that were modified in ICR^Δ^ [44]: 5′- AACA AGGG AACG GATG CTAC CG -3′ and 5′- GCAA TATG TAGT ATTG TACT GCCA CCAC -3′, lack of a 506 bp amplicon identified ICR^Δ.Δ^ animals. For *Cdkn1c*, the null allele was identified using primers specific for the selection cassette using in gene targeting: 5′- CTCA GAGG CTGG GAAG GGGT GGGT C -3′, within the mouse ‘phosphoglycerate kinase 1’ (*Pgk1*) promoter, and 5′- ATAC TTTC TCGG CAGG AGCA AGGT G -3′, within the *neo* coding sequence, 520 bp amplicon.

### Northern Blots

Fetuses were genotyped, and total RNA recovered using RNAzol (Tel-Test) after homogenization of the total fetus minus the head. Probes for *Igf2* and ‘glyceraldehyde-3-phosphate dehydrogenase’ (*Gapdh*) RNA were as previously described [67]. The *Cdkn1c* probe was made by RT-PCR using primers; 5′- GCCG GGTG ATGA GCTG GGAA -3′ and 5′- AGAG AGGC TGGT CCTT CAGC -3′, 221 bp amplicon. Northern blots were performed with ^32^P radiolabelled probes as described previously [68]. The three probes were hybridized independently to the same blots after stripping. Radioactivity of bands was quantitated using a Typhoon PhosphorImager (Molecular Dynamics). For each lane, values for *Igf2* and *Cdkn1c* were normalized to the *Gapdh* value.
